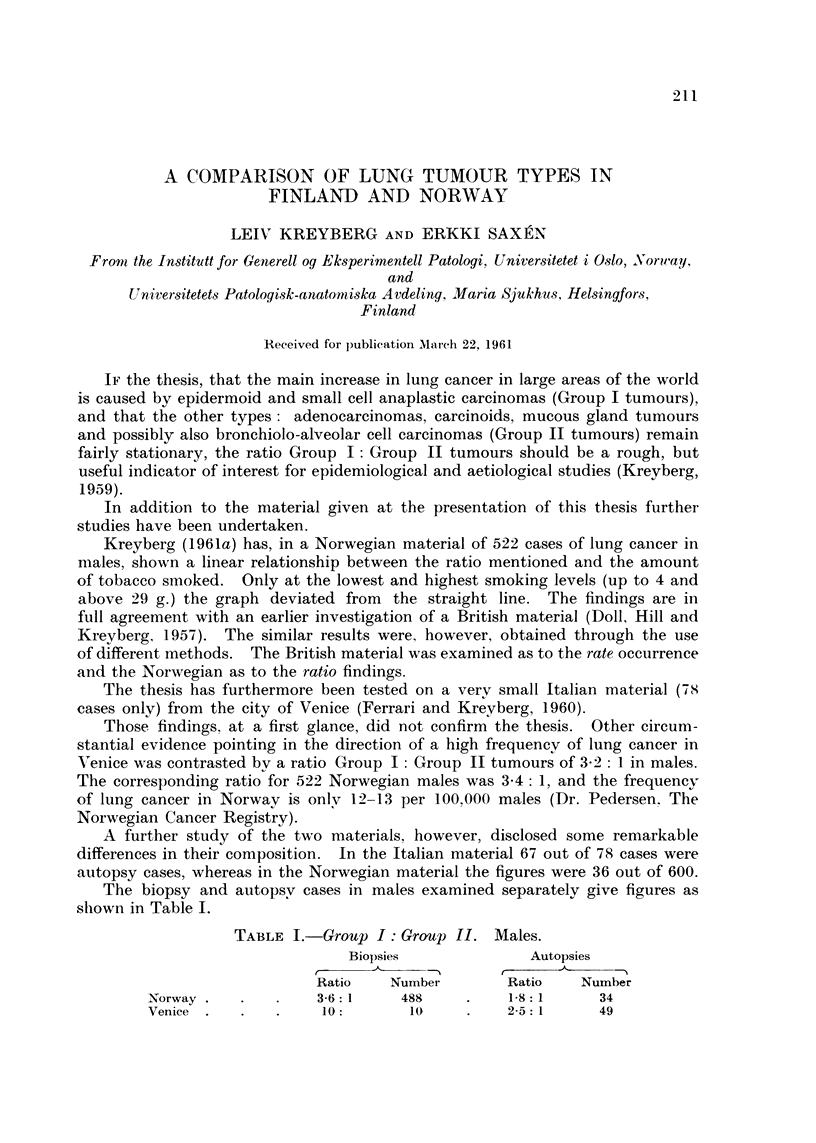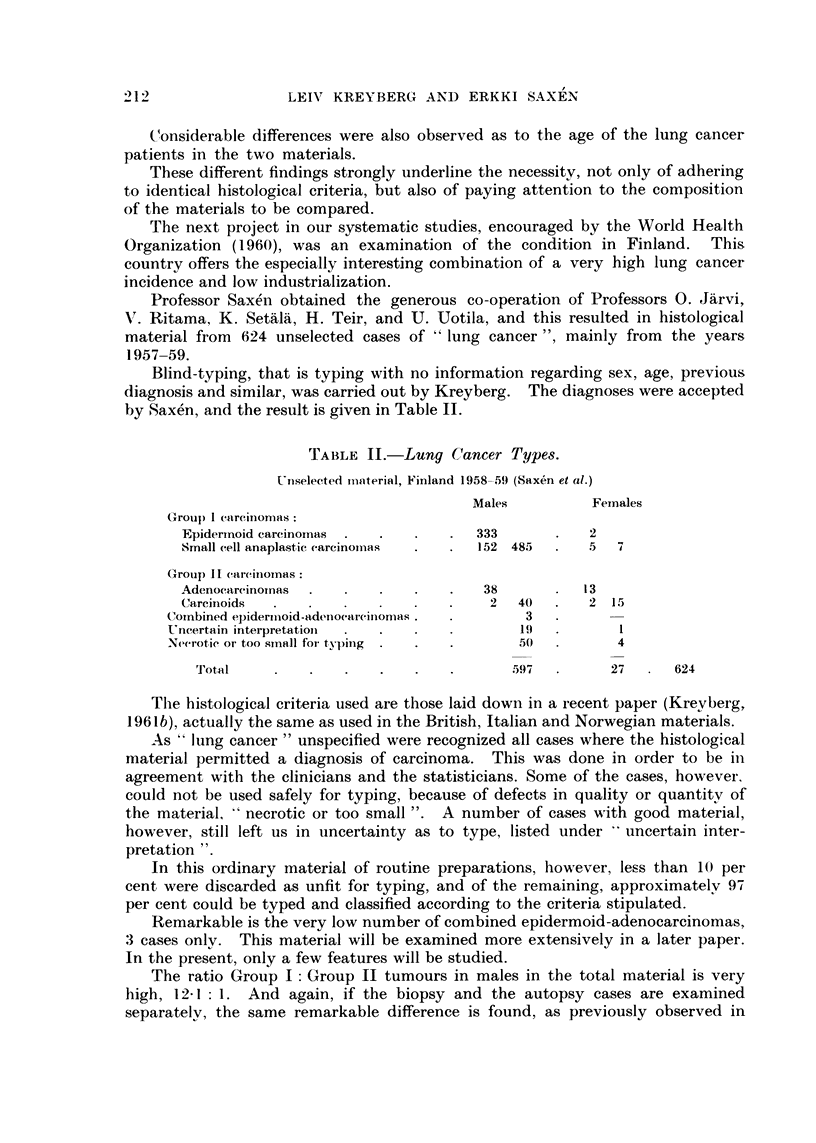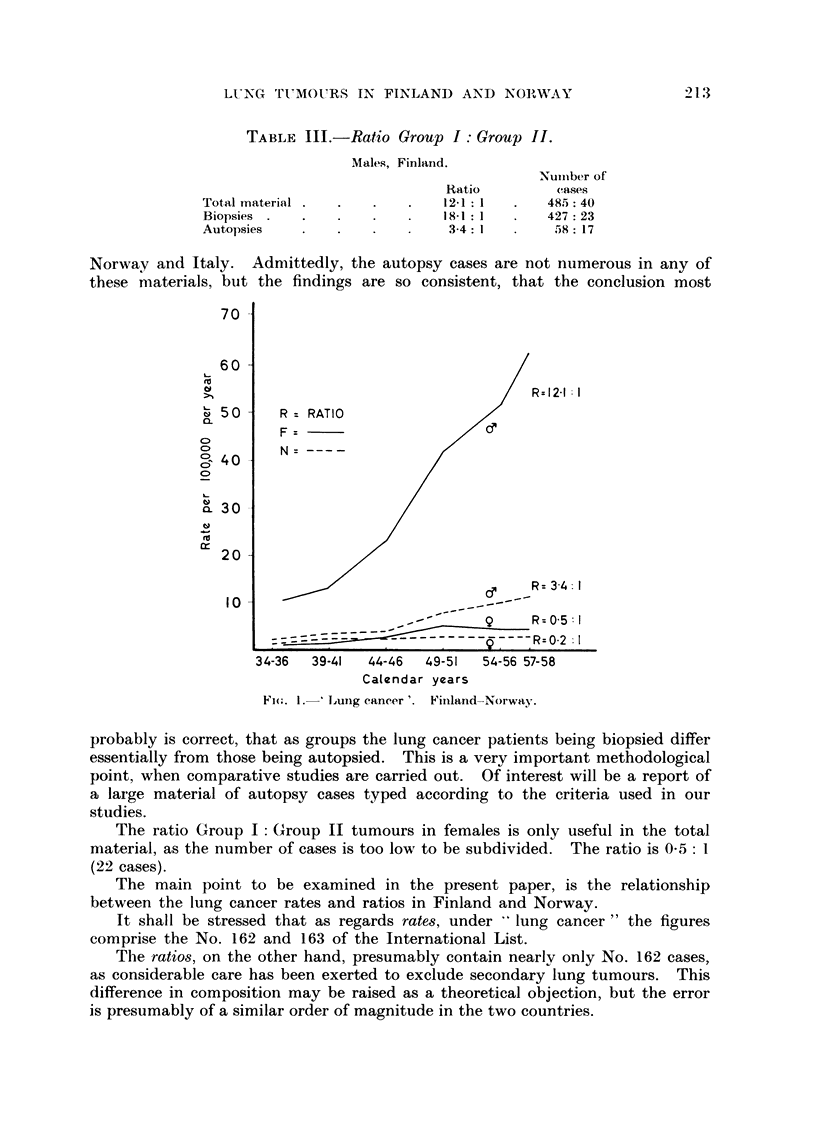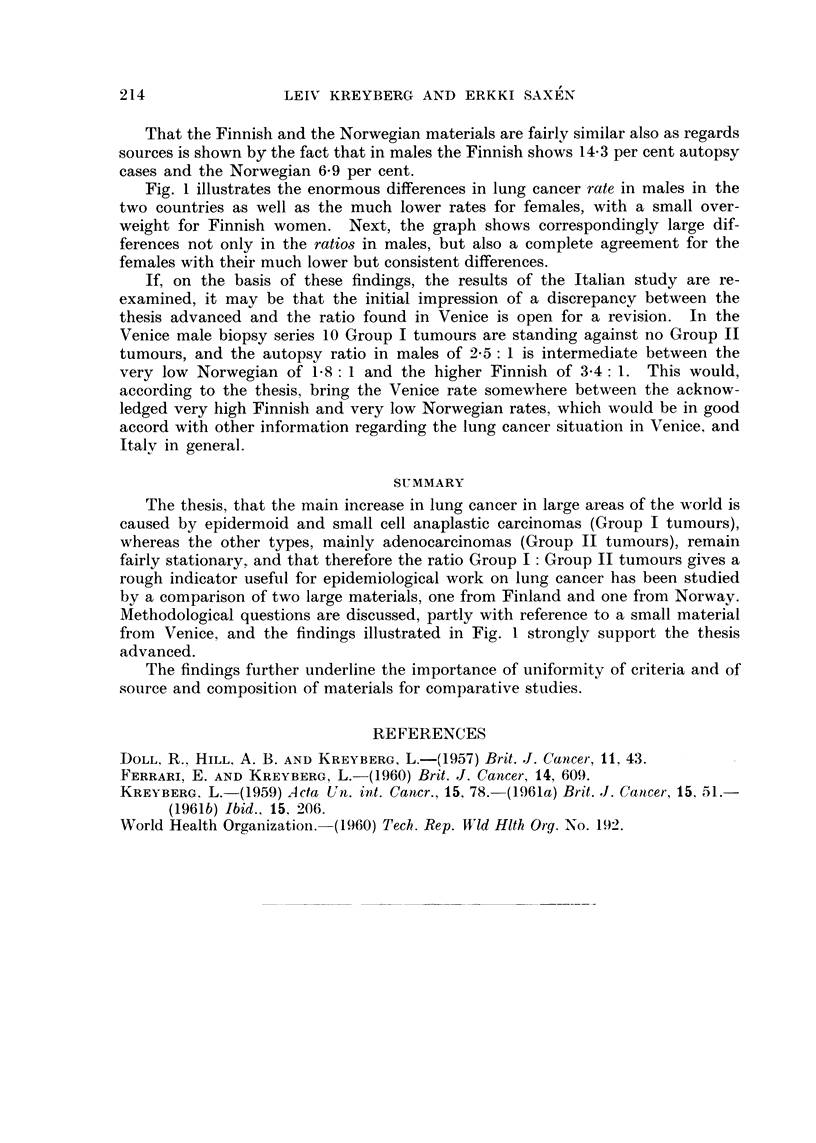# A Comparison of Lung Tumour Types in Finland and Norway

**DOI:** 10.1038/bjc.1961.26

**Published:** 1961-06

**Authors:** Leiv Kreyberg, Erkki Saxén


### A COMPARISON OF LUNG TUMOUR TYPES IN FINLAND AND NORWAY

LEIV KREYBERG AND ERKKI SAXÉN

*From the Institutt for Generell og Eksperimentell Patologi, Universitetet i Oslo, Norway, and Universitetets Patologisk-anatomiska Avdeling, Maria Sjukhus, Helsingfors, Finland*

Received for publication March 22, 1961

IF the thesis, that the main increase in lung cancer in large areas of the world is caused by epidermoid and small cell anaplastic carcinomas (Group I tumours), and that the other types : adenocarcinomas, carcinoids, mucous gland tumours and possibly also bronchiolo-alveolar cell carcinomas (Group II tumours) remain fairly stationary, the ratio Group I : Group II tumours should be a rough, but useful indicator of interest for epidemiological and aetiological studies (Kreyberg, 1959).

In addition to the material given at the presentation of this thesis further studies have been undertaken.

Kreyberg (1961*a*) has, in a Norwegian material of 522 cases of lung cancer in males, shown a linear relationship between the ratio mentioned and the amount of tobacco smoked. Only at the lowest and highest smoking levels (up to 4 and above 29 g.) the graph deviated from the straight line. The findings are in full agreement with an earlier investigation of a British material (Doll, Hill and Kreyberg, 1957). The similar results were, however, obtained through the use of different methods. The British material was examined as to the *rate* occurrence and the Norwegian as to the *ratio* findings.

The thesis has furthermore been tested on a very small Italian material (78 cases only) from the city of Venice (Ferrari and Kreyberg, 1960).

Those findings, at a first glance, did not confirm the thesis. Other circumstantial evidence pointing in the direction of a high frequency of lung cancer in Venice was contrasted by a ratio Group I : Group II tumours of 3·2 : 1 in males. The corresponding ratio for 522 Norwegian males was 3·4 : 1, and the frequency of lung cancer in Norway is only 12–13 per 100,000 males (Dr. Pedersen, The Norwegian Cancer Registry).

A further study of the two materials, however, disclosed some remarkable differences in their composition. In the Italian material 67 out of 78 cases were autopsy cases, whereas in the Norwegian material the figures were 36 out of 600.

The biopsy and autopsy cases in males examined separately give figures as shown in Table I.

TABLE I.—*Group I : Group II.* Males.

| | Biopsies | | Autopsies | |
|---|---|---|---|---|
| | Ratio | Number | Ratio | Number |
| Norway | 3·6 : 1 | 488 | 1·8 : 1 | 34 |
| Venice | 10 : | 10 | 2·5 : 1 | 49 |

Considerable differences were also observed as to the age of the lung cancer patients in the two materials.

These different findings strongly underline the necessity, not only of adhering to identical histological criteria, but also of paying attention to the composition of the materials to be compared.

The next project in our systematic studies, encouraged by the World Health Organization (1960), was an examination of the condition in Finland. This country offers the especially interesting combination of a very high lung cancer incidence and low industrialization.

Professor Saxén obtained the generous co-operation of Professors O. Järvi, V. Ritama, K. Setälä, H. Teir, and U. Uotila, and this resulted in histological material from 624 unselected cases of " lung cancer ", mainly from the years 1957–59.

Blind-typing, that is typing with no information regarding sex, age, previous diagnosis and similar, was carried out by Kreyberg. The diagnoses were accepted by Saxén, and the result is given in Table II.

TABLE II.—*Lung Cancer Types.*

Unselected material, Finland 1958–59 (Saxén *et al.*)

| | Males | | Females | | |
|---|---|---|---|---|---|
| Group I carcinomas : | | | | | |
| Epidermoid carcinomas | 333 | | 2 | | |
| Small cell anaplastic carcinomas | 152 | 485 | 5 | 7 | |
| Group II carcinomas : | | | | | |
| Adenocarcinomas | 38 | | 13 | | |
| Carcinoids | 2 | 40 | 2 | 15 | |
| Combined epidermoid-adenocarcinomas | | 3 | | — | |
| Uncertain interpretation | | 19 | | 1 | |
| Necrotic or too small for typing | | 50 | | 4 | |
| Total | | 597 | | 27 | 624 |

The histological criteria used are those laid down in a recent paper (Kreyberg, 1961*b*), actually the same as used in the British, Italian and Norwegian materials.

As " lung cancer " unspecified were recognized all cases where the histological material permitted a diagnosis of carcinoma. This was done in order to be in agreement with the clinicians and the statisticians. Some of the cases, however, could not be used safely for typing, because of defects in quality or quantity of the material, " necrotic or too small ". A number of cases with good material, however, still left us in uncertainty as to type, listed under " uncertain interpretation ".

In this ordinary material of routine preparations, however, less than 10 per cent were discarded as unfit for typing, and of the remaining, approximately 97 per cent could be typed and classified according to the criteria stipulated.

Remarkable is the very low number of combined epidermoid-adenocarcinomas, 3 cases only. This material will be examined more extensively in a later paper. In the present, only a few features will be studied.

The ratio Group I : Group II tumours in males in the total material is very high, 12·1 : 1. And again, if the biopsy and the autopsy cases are examined separately, the same remarkable difference is found, as previously observed in

TABLE III.—*Ratio Group I : Group II.*

Males, Finland.

| | Ratio | Number of cases |
|---|---|---|
| Total material | 12·1 : 1 | 485 : 40 |
| Biopsies | 18·1 : 1 | 427 : 23 |
| Autopsies | 3·4 : 1 | 58 : 17 |

Norway and Italy. Admittedly, the autopsy cases are not numerous in any of these materials, but the findings are so consistent, that the conclusion most

FIG. 1.—' Lung cancer '. Finland–Norway.

probably is correct, that as groups the lung cancer patients being biopsied differ essentially from those being autopsied. This is a very important methodological point, when comparative studies are carried out. Of interest will be a report of a large material of autopsy cases typed according to the criteria used in our studies.

The ratio Group I : Group II tumours in females is only useful in the total material, as the number of cases is too low to be subdivided. The ratio is 0·5 : 1 (22 cases).

The main point to be examined in the present paper, is the relationship between the lung cancer rates and ratios in Finland and Norway.

It shall be stressed that as regards *rates*, under " lung cancer " the figures comprise the No. 162 and 163 of the International List.

The *ratios*, on the other hand, presumably contain nearly only No. 162 cases, as considerable care has been exerted to exclude secondary lung tumours. This difference in composition may be raised as a theoretical objection, but the error is presumably of a similar order of magnitude in the two countries.

That the Finnish and the Norwegian materials are fairly similar also as regards sources is shown by the fact that in males the Finnish shows 14·3 per cent autopsy cases and the Norwegian 6·9 per cent.

Fig. 1 illustrates the enormous differences in lung cancer *rate* in males in the two countries as well as the much lower rates for females, with a small overweight for Finnish women. Next, the graph shows correspondingly large differences not only in the *ratios* in males, but also a complete agreement for the females with their much lower but consistent differences.

If, on the basis of these findings, the results of the Italian study are re-examined, it may be that the initial impression of a discrepancy between the thesis advanced and the ratio found in Venice is open for a revision. In the Venice male biopsy series 10 Group I tumours are standing against no Group II tumours, and the autopsy ratio in males of 2·5 : 1 is intermediate between the very low Norwegian of 1·8 : 1 and the higher Finnish of 3·4 : 1. This would, according to the thesis, bring the Venice rate somewhere between the acknowledged very high Finnish and very low Norwegian rates, which would be in good accord with other information regarding the lung cancer situation in Venice, and Italy in general.

### SUMMARY

The thesis, that the main increase in lung cancer in large areas of the world is caused by epidermoid and small cell anaplastic carcinomas (Group I tumours), whereas the other types, mainly adenocarcinomas (Group II tumours), remain fairly stationary, and that therefore the ratio Group I : Group II tumours gives a rough indicator useful for epidemiological work on lung cancer has been studied by a comparison of two large materials, one from Finland and one from Norway. Methodological questions are discussed, partly with reference to a small material from Venice, and the findings illustrated in Fig. 1 strongly support the thesis advanced.

The findings further underline the importance of uniformity of criteria and of source and composition of materials for comparative studies.

### REFERENCES

Doll, R., Hill, A. B. and Kreyberg, L.—(1957) *Brit. J. Cancer*, **11**, 43.

Ferrari, E. and Kreyberg, L.—(1960) *Brit. J. Cancer*, **14**, 609.

Kreyberg, L.—(1959) *Acta Un. int. Cancr.*, **15**, 78.—(1961*a*) *Brit. J. Cancer*, **15**, 51.—(1961*b*) *Ibid.*, **15**, 206.

World Health Organization.—(1960) *Tech. Rep. Wld Hlth Org.* No. 192.